# ‘Wala kaming alam sa stroke care’ (we don’t know anything about stroke care): experiences of care providers delivering stroke rehabilitation care in the Philippines

**DOI:** 10.1080/16549716.2026.2682047

**Published:** 2026-06-05

**Authors:** Fiona Leggat, Sarah Ann Buckingham, Aira Patrice Rueda Ong, Bridie Kent, Nena Marie Santos, Annah Teves, Vergil Boac, Paula Melizza Valera, Elda Anota, Maria Mercedes Barba, Myrna Estrada, Neil Bryan Pita, Alyssa Dar Juan, Lorraine Faeldon, Fiona Jones

**Affiliations:** a Population Health Research Institute, School of Health and Medical Sciences, City St George’s University of London, London, UK; b School of Nursing and Midwifery, Faculty of Health, University of Plymouth, Plymouth, Devon, UK; c Evelyn D. Ang - Institute of Biomedical Engineering and Health Technologies, De La Salle University, Manila, Philippines; d Bridges Self-Management, London, UK

**Keywords:** Stroke rehabilitation, low-middle-income country, healthcare professionals, qualitative, service provision

## Abstract

**Background:**

In the Philippines, rising stroke prevalence and healthcare inequalities present a concerning outlook. While acute stroke care is prioritised, rehabilitation remains undervalued. The experiences of healthcare providers who deliver stroke support are also under-researched.

**Objective(s):**

Our study aimed to explore the experiences and needs of care providers across multiple regions of the Philippines to inform the development of stroke support initiatives and programmes.

**Methods:**

Forming part of the Tulong, Ugnayan ng Lingap At gabaY (TULAY) project, a qualitative descriptive design involving semi-structured interviews was used. Care providers with professional or voluntary community healthcare service delivery roles were recruited. Data were analysed using reflexive thematic analysis.

**Results:**

Seventeen participants across five regions were interviewed between June and September 2024. Three high-level themes were constructed: 1) inequitable stroke care and rehabilitation services, 2) barriers to the provision and access of stroke services and 3) desires, needs and opportunities for improvement.

**Conclusions:**

Traditionally multi-level (e.g. national, community) approaches offer solutions to enhancing stroke rehabilitation in the Philippines. However, due to the devolved healthcare system, community-based initiatives, cognisant of local contexts and built together with stroke survivors, offer a promising solution to improve the lives of Filipinos following stroke.

## Paper Context

- **Main findings:** National, community, and individual level barriers including geography, finance, resources, politics and knowledge continue to foster stroke rehabilitation inequities across the Philippines.
- **Added knowledge:** The devolved and fragmented healthcare system in the Philippines is challenging for the upskilling and education of those delivering stroke services which can lead to limited knowledge of stroke rehabilitation and the quality and delivery of care in local communities.
- **Global health impact for policy and action:** Government action, prioritisation, and policy formation is necessary to mandate stroke rehabilitation services across the Philippines, but community-based rehabilitation offers a practical bottom-up solution to improving the knowledge and wellbeing of stroke survivors in the interim.

## Background

Amid a rising stroke burden across Low- and Middle-income countries (LMICs), the Philippines has seen one of the largest global increases in Intra Cerebral Haemorrhage (ICH) over the past three decades [1,2]. In 2021, stroke was the second leading cause of death in the country [2]. Demographically, higher incidences of haemorrhagic strokes are found in men, while women are more likely to experience ischemic strokes [3]. The country has a high haemorrhagic stroke incidence, especially among younger adults [4], and there are considerable rural-urban disparities in the care available to stroke survivors and household carers. Currently, 67% of neurologists work in urban areas, with rural patients facing delayed or suboptimal acute treatment, and barriers such as transportation and stroke awareness [5,6]. Stroke prevalence may also be undercounted due to limited data infrastructure and suspected underreporting in rural areas, where limited imaging and data collection suppress true incidence estimates [7].

The Philippines’ archipelagic geography of 7641 islands [8] and its devolved healthcare system complicates access to stroke care. Combined with rural-urban disparities and low public awareness, this has strained resources. Public health is managed by the Department of Health and PhilHealth, a national insurance scheme designed to enable reimbursement for health services under the Universal Health Care (UHC) law [9]. However, the system relies upon devolved governance to local government units (LGUs). The country comprises nested administrative structures with 18 regions, 82 provinces, approximately 149 cities, and 1,493 municipalities [10]. Barangays are the smallest geographical subdivision, nested within municipalities/cities, and are the first level of government contact responsible for managing health. There are currently more than 42,000 Barangays [10]. Qualified healthcare professionals in the Philippines are mostly physicians, health officers (GPs), nurses, midwives and social welfare officers. However, at the community level, stroke care largely depends on volunteer Barangay Health Workers (BHWs), the most grassroots-level position [11]. The role of BHWs in stroke survivor support is limited, and in similarity with healthcare professionals, little is known about their experiences delivering stroke services. A 2022 review identified poor community stroke awareness, a severe shortage of stroke-trained professionals, and the need for community-level education and training in areas where BHWs already serve [12]. With limited stroke rehabilitation facilities, partially due to financial and geographical constraints [13], BHWs and community nurses, and where available physical and occupational therapists, are required to coordinate home-based rehabilitation services and ease the burden on family caregivers [14].

This paper presents one of the first qualitative studies which sought to explore the experiences, challenges, desires and recommendations of care providers supporting stroke survivors across urban and rural settings in the Philippines [15]. This forms part of the Tulong, Ugnayan ng Lingap At gabaY (TULAY) project, which has also explored the experiences and unmet needs of stroke survivors and their carers, to more fully understand the nature and quality of existing stroke rehabilitation and care services nationwide [15,16]. These in-depth qualitative studies will provide qualitative data to complement and build on the knowledge of stroke services gained in a previous national survey [17]. The findings will be used to inform the co-design of a culturally relevant stroke support programme in the Philippines, tailored to the needs of all stakeholders, including care providers.

## Methods

### Study design and governance

The study was underpinned by ontological relativism (i.e. reality is multiple) and epistemological constructionism (i.e. knowledge is subjective and constructed) and used a descriptive qualitative design. Further details of the study design are published elsewhere [15]. The manuscript follows the Consolidated Criteria for Reporting Qualitative Research (COREQ) checklist [18]. The TULAY research team comprised colleagues from the UK and the Philippines.

Ethical approval was granted by the Philippines Department of Health Single Joint Research Ethics Board (SJREB) and the University of Plymouth Faculty of Health Research Ethics and Integrity Committee. The study was performed in accordance with the principles stated in the Declaration of Helsinki [19]. All participants were able to read the study information sheet and ask questions prior to being asked to give their written informed consent to participate. However, owing to the geographical spread of participants, a flexible consent process was used. This meant participants in isolated areas, without internet access, could give audio-recorded verbal consent for the study.

### Recruitment & sampling

Care providers were recruited from an existing TULAY project database which contained details of individuals who had participated in earlier project stages and expressed a willingness to participate in follow-up interviews [15]. Snowball sampling through participant community networks and contacts of the wider TULAY team was also used. The study aimed to recruit a diverse sample, with representation across all sites, and reach data saturation.

Care providers were described as individuals in formal/professional or voluntary/community roles (e.g. BHWs), who had supported at least one person with stroke in the past 12 months. This group included qualified healthcare professionals (e.g. clinicians, nurses) and those occupying community-based health support roles (e.g. BHWs, nurses, midwives, social welfare officers, support workers) or governmental health officer roles (e.g. municipal health officers). Multiple care provider roles were recruited using opportunistic sampling to gather representation across the range of roles involved in delivering community-level stroke care.

**Figure 1.**
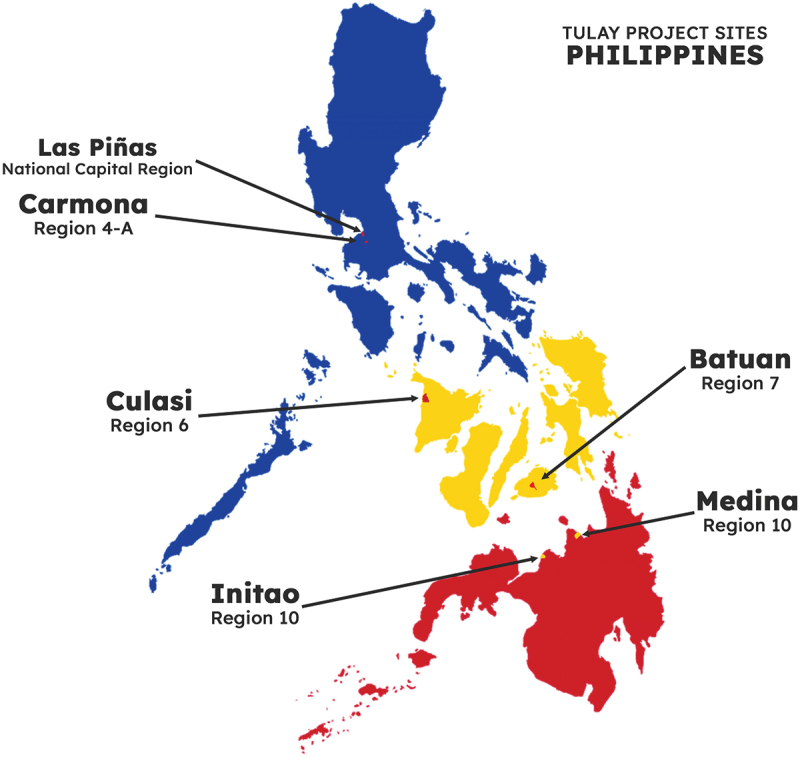
Geographical spread of participants across the Philippines.

Recognising inequalities across the Philippines [20], we aimed to recruit a diverse participant group from across five Philippines regions. Three key purposive criteria were used to sample care providers localities: socio-economic level, urban/rural, and geographical area (region) [15]. These regions represent all three primary geographical divisions of the archipelago, including two in Luzon, the largest and northernmost island group (National Capital Region/Region 4-A), two in the Visayas, the central part of the archipelago (Western Visayas, Region 6/ Central, Region 7), and one region in Mindanao, located in the south (Northern Mindanao, Region 10) (see Figure 1). These regions were chosen due to their previous participation in the wider TULAY project [17], established researcher/clinician connections with LGUs, and their accessibility, in consideration of travel, time and financial constraints.

### Data collection

Semi-structured interviews were undertaken with care providers in the preferred dialect of the interviewee (e.g. English, Tagalog, Cebuano, Bisaya), by one or two interviewers (if two interviewers completed the interview, one asked the questions while the other compiled field notes) (*n* = 2 male, 7 female) between June and September 2024. Interviewers were from the Philippine team, who spoke the language and understood the healthcare system. All held researcher contracts, had received qualitative research and narrative interview training, and had no prior relationship with any of the participants. The Philippine team’s fluency in local dialects and knowledge of regional services and/or stroke services was sought to build trust with participants and encourage them to speak freely. In regular team meetings, interviewers were encouraged to be reflexive and mindful of any power imbalances which may impact participant disclosure or their use of follow-up questions and prompts.

Interviews were held face-to-face at a time, date and location of participants’ choice, considering practicality, accessibility, privacy, safety and security factors. All interviews were undertaken at care providers’ workplaces (e.g. barangay health centres, hospitals). Only care providers and interviewers were present during interviews. All interviews were audio recorded, and field notes were taken by interviewers to record contextual observations and reflections. During the close of each interview, participants’ responses were summarised for clarification and interpretation.

Topic guides were developed with support from patient and public members in the wider TULAY project, piloted with two care providers, and used to guide the interview structure. Topics included exploration of existing stroke services, care providers’ experiences delivering stroke rehabilitation and services, barriers and enablers to stroke services and rehabilitation, and recommendations for service improvement, the TULAY programme and other care providers (see Supplementary File 1).

### Data analysis & rigor

All interviews were transcribed verbatim in their original dialect. All identifiable information was removed during this process (e.g. individual names, place names) and transcripts were labelled with each participant’s alphanumeric identifier (Care Provider no.) to ensure anonymity. All transcripts were translated into English prior to analysis.

Data were analysed using thematic analysis [21]. A small q approach was taken combining codebook and inductive, reflexive elements [22,23]. Initially, transcripts were uploaded to NVivo 14 software; four transcripts were read and coded by two members of the research analysis team (SB, APRO) to develop a preliminary framework. The remaining transcripts were coded by four additional researchers, including three from the Philippine team (FL, VB, AT, NMS). Transcripts were assigned inductive and deductive codes based upon the interview topic guide and data-driven content. To facilitate simultaneous coding by the UK and Philippine teams, the Collaboration Cloud feature of NVivo 14 [24] was used.

Multiple strategies were used to enhance the rigor and trustworthiness of the analysis. During coding, all coders documented their observations and reflexive notes. Two transcripts were blindly second coded by a member of the team (FL), and using the coding comparison query within Nvivo, inter-coder agreement reached approximately 84%. This score accounts for differences in contextual interpretation, but similar meaning in the data. Codes were debated and discussed between the coders who acted as critical friends with one another during weekly analysis meetings and secure chat groups. This enabled ‘insiders’ (Philippines team) and ‘outsiders’ (UK team) to explore different interpretations based on their positionality. The Philippine team had an innate cultural understanding of Filipino social norms, and traditions which aided their interpretation. The final codebook is shown in Supplementary File 2. Descriptive summaries were created following the refinement of codes. NVivo queries were used to explore geographical and urban/rural differences and nuances within the data.

To develop themes with descriptive summaries, Xmind AI, an online mind-mapping and presentation software [25] was used to visually organise codes and explore any relationships. The lead author developed a visual thematic map which was refined collaboratively between the UK and Philippine coders during weekly team meetings to maintain cultural sensitivity and enhance rigor. Themes were described and quotes attached to provide rich meaning and illustration of the data. Some quotes were translated back into Filipino dialects to reflect the spoken words of participants. To enhance the trustworthiness of the theme development, initial theme summaries were shared with a Luzon-based care provider and care provider co-authors (PMV, EA, MMB, ME). This group confirmed that the summaries aligned with their own and colleagues’ experiences. A full audit trail, including coding decisions and the analysis process, was maintained in NVivo.

## Results

### Participant characteristics

Seventeen care providers were interviewed across the five regions. Six interviews were conducted in Tagalog, one in English, and ten in Cebuano/Bisaya. Interviewees’ demographic profiles are shown in Table 1. Interview durations ranged from 18 minutes to 68 minutes (*M* = 40 minutes). There was no participant drop out.

**Table 1.** Demographics and characteristics of participants.

	Care providers (N = 17)
**Age** (years) Mean (SD) Range	41.9 (12.5) 27 to 65
**Gender** (%) Male Female	4 (24) 13 (76)
**Ethnicity** (%) Tagalog Illonggo/Karay-a Cebuano/Binisaya/Bisaya/Bol anon	6 (35) 1 (6) 10 (59)
**Region** (%) Las Piñas (National Capital Region) Carmona (Region 4) Culasi (Region 6) Batuan (Region 7) Initao/Medina (Region 10)	3 (18) 3 (18) 1 (6) 4 (24) 6 (36)
**Urban or rural residence** (%) Urban Rural	6 (35) 11 (65)
**Socioeconomic status** (%) Low Middle/high	7 (41) 10 (59)
**Occupation** (%) Barangay Health Worker (BHW) Medical Doctor Midwife Nurse Occupational Therapist	4 (24) 1 (6) 3 (18) 8 (47) 1 (6)

Note: Percentages may not total 100 due to rounding. SD = Standard Deviation. Urban/rural residence was self-reported in a questionnaire completed prior to participation. Classification of socioeconomic status was based on self-reported data on education and income; where these data were not available, regional indicators of poverty incidence were used as an estimate, i.e. lower than national average = middle/high socioeconomic status, higher than national average = low socioeconomic status (as defined by the Philippine Statistics Authority [26].

### Interview themes

**Figure 2.**
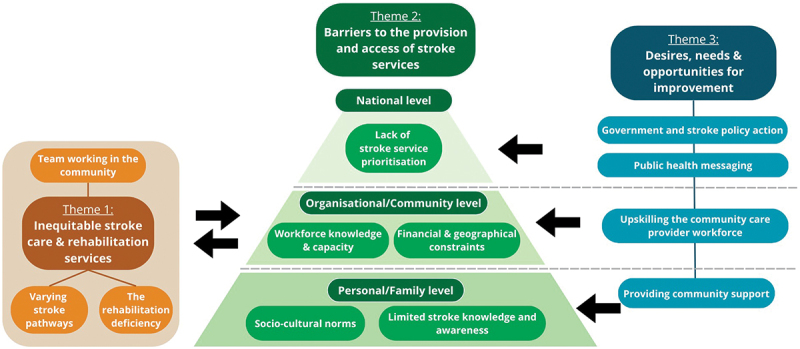
Three key themes and their interactions.

Three key themes were constructed from the data, each with several sub-themes (see Figure 2).

#### Theme 1: Inequitable stroke care and rehabilitation services

Across the Philippines, inequities in stroke care and rehabilitation were apparent. Overall, urban areas were considered to provide higher levels of stroke care provision, compared to rural areas with more sparse provision. This theme, specifically related to the content of service provision, is comprised of three sub-themes.

##### Varying stroke pathways

Care providers spoke about the prevalence of different stroke pathways which included the diagnostic process, and onward referral for aftercare. Throughout the country, stroke diagnosis occurred at the community level from BHWs, nurses and doctors who checked vital markers in those suspected of experiencing a stroke. After diagnosis, referrals to hospital could be made if a stroke survivor’s needs exceeded care providers’ professional expertise within local health centres. Referrals could be made to other medical staff (e.g. doctors) in the local Rural Health Unit (RHU) or City Health Office (CHO), as well as hospitals or specialist services in severe cases. Hospitals differed in their focus, capability, and location (e.g. Level 1 [basic care, initial treatment, general medicine, minor surgeries], Level 2 [broader range of services], Level 3 [specific, specialised, more complex health conditions]). Hospitals offering more comprehensive support tended to focus on specific medical-surgical specialisms (e.g. orthopaedics, paediatrics), were most expensive and were usually in urban areas (e.g. National Capital Region). Findings illustrated large inequities in hospital distribution across the Philippines and regional variability in the referral process was apparent. Across some rural areas (e.g. Medina), a pathway was absent, leaving care providers reliant on their own experience. Yet, mainly in urban areas, care providers described a clear, structured hierarchy escalating cases to higher-level facilities (e.g. Carmona, Batuan):

The referral system here is fast. It starts with us here [Barangay Health Centre]. Then we forward it to the CHO [City Health Office]. If they are not available, they automatically forward/transfer to the government hospital, and they will refer to where they can (if it can’t be handled there). (CP005)

##### Team working in the community

Care providers described collaborative working in local communities. Through their regular presence and provision of basic prevention services, initial assessments, home visits, medication delivery and care monitoring, BHWs were regarded as the first point of contact and those most knowledgeable of their communities:

They [BHWs] do home visits more regularly because I’m handling other programs … they certainly are my frontliners… It’s their community, they know the community. (CP015)

BHWs occasionally gave basic health education to stroke survivors and their families during home visits. However, the frequency of visits ranged from once a week (e.g. Medina), to every other month (e.g. Batuan), and in some cases were only offered if stroke survivors were unable to access a health centre (e.g. Culasi).

To support BHWs and provide basic community stroke care, the local networks of doctors, nurses, and midwives were considered vital. Coordination and communication between professions (e.g. nurses, doctors, midwives) when available, and BHWs, supported the delivery of stroke care through the top-down sharing of knowledge. Such teamwork and supportive discussion for care management, including diagnosis and referral, were perceived positively by care providers, especially BHWs.

##### The rehabilitation deficiency

Care providers emphasised an overwhelming lack of community rehabilitation services. Even in some urban communities (e.g. Carmona, Batuan), there were no designated stroke-specific rehabilitation facilities. This meant stroke survivors could be referred to the CHO, hospitals in other cities, or left to rely on home care from BHWs untrained in stroke:

There is no stroke care available in our area. There’s stroke care in hospitals, and as far as I can remember, the General Hospital has, but here, there’s none. (CP003)

Some communities had visits from physiotherapists, and whilst one BHW had received community-based rehabilitation training, these occurrences were considered rare: “*We are the only one who provide this service. I am the only one who visits’”*(CP014).

With an absence of free services, private clinics provided stroke rehabilitation in some urban, and occasional rural areas. Private rehabilitation was often unfunded and expensive, but often the only access to physiotherapy: “*those who receive PT (physiotherapy) are those who go to private physicians”* (CP009). Private services were not always local, and thus, challenging for stroke survivors to access even on a one-off basis due to resource burden (e.g. finance, transport).

#### Theme 2: Barriers to the provision and access of stroke services

This theme is presented in three sub-themes to illustrate factors at different levels of Philippine context: national, regional/community and personal/family.

##### Lack of stroke service prioritisation

The lack of structured, formal stroke guidelines, protocols, and funding was considered challenging by care providers. Unlike other diseases (e.g. tuberculosis), or immunizations, stroke was perceived to lack prioritisation at the national level and thus impacting funding and resources at regional and community levels. Without a national mandate to prioritise stroke, care providers described how the allocation of stroke care funding was left to the priorities of LGUs:

We have a substantial health budget, but no specific funding allocated for stroke care. National regulations don’t mandate or compel the LGU to do that. For instance, the nutrition program or the HIV program has a specific law that requires the LGU to set aside a certain amount for that. But for stroke rehab, there’s nothing. (CP012)

With a lack of national guidelines, care providers felt they had no best-practice approach and instead were reliant on research and guidance from other countries to inform their stroke rehabilitation. Yet, interventions and protocols from the west (e.g. Europe/USA) were not always considered appropriate to the national culture or resource provision:

Most of my protocols are based in another country. I don’t see anything that is Philippine-based … so another big factor is the cultural difference. The activities they provide, we don’t have. We have no resources. We have to modify so the intervention provided is somehow the same. (CP002)

##### Workforce knowledge and capacity

Care providers’ busy workloads and limited knowledge, skills and confidence in stroke rehabilitation were felt to inhibit provision. Care providers in urban and rural municipalities and barangays juggled the delivery of multiple national healthcare priorities (e.g. immunization, prenatal care, family planning, and emergency responses), with only limited staff. Retention of staff was considered a problem, with more attractive opportunities abroad (e.g. Europe). The shortage of healthcare professionals, and sometimes complete absence of therapy-trained staff (e.g. physiotherapists) in rural areas meant stroke rehabilitation was often not delivered or offered. The national healthcare priorities created demanding workloads which restricted care providers’ capacity to provide support for stroke survivors:

At times as a BHW, we are truly loaded with work. We truly have so much work, and there are already plenty of activities (CP004).

Most care providers also described a lack of any formal stroke rehabilitation training. Some had received minimal stroke prevention training, or a brief introduction during medical training, but beyond this, care providers emphasised challenges accessing education and training. BHWs were considered to have more time and opportunity to support stroke survivors, especially in rural areas, yet lacked knowledge and confidence to deliver rehabilitation. Instead, BHWs referred survivors to doctors in regional hospitals who had some knowledge, but less available time. Overall, most care providers felt their stroke knowledge and skills were insufficient:

I: Do you think the BHWs have enough knowledge to help [stroke survivors]?

Nobody has that knowledge, no one. Most of the BHWs have not attended training. (CP014)

##### Financial and geographical constraints

The sparse nature of public healthcare services was considered to heighten geographical and financial barriers for stroke survivors. With limited funding for transportation, or for home visits, stroke survivors were perceived to be left with burdensome journeys and obstacles restricting healthcare access. Care providers suggested that public hospitals presented more financially viable options, compared to private clinics, but often posed the greatest geographical hurdles requiring substantial travel. Even in urban areas with more transportation and public services, stroke survivors could be constrained by physical mobility issues, notwithstanding general cultural challenges including extreme weather systems, and unstable terrain. Some barangays provided ambulance services, but these could be unreliable or unavailable, especially in rural or GIDAs (Geographically Isolated and Disadvantaged Areas) without access roads:

I: What are the most common reasons why patients cancel?

Usually because of rains and the typhoons. Others because of the transportation. Others because their helper is not available. But mostly it’s the transport because almost all of our patients in the hospital, they rely on the ambulance of the barangay. They cannot afford to take a taxi because it’s hassle and it’s hard for them to walk. Usually, they rely on the ambulance and sometimes when it is not available, they can’t go. (CP002)

Stroke survivors were perceived to rely on family or neighbours for practical support accessing healthcare. Care providers described how few had insurance, or access to financial government initiatives. The financial burden was not just related to rehabilitation, but the entire stroke pathway. Cost was perceived to make some services and medication completely inaccessible for low-income stroke survivors, only enhancing the risk of further post-stroke complications: “*sometimes there is no free medicine, and the patient has no money to buy. The others, they stop medicine, and the sickness gets worse”* (CP006).

##### Socio-cultural norms

The culture, beliefs and motivation of stroke survivors were perceived to impact their drive to source and attend stroke care appointments. Stroke survivors were considered to prioritise their family’s needs above their own. This meant they prioritised food and resources for others, above wanting to ask for help with paying for or travelling to access healthcare services. Therefore, those with bigger, local, and more affluent families were considered more able to access healthcare services, as well as family support, compared to those with smaller, less-affluent families who had competing priorities (e.g. employment).

Stroke survivors’ motivation to recover was also suggested to influence care access. Whilst some were considered keen and focused on recovery, others were considered passive, with a learned helplessness arising from low self-esteem, or a culture of waiting for and/or expecting to be given a ‘fix’ by a care provider. In some rural areas, care providers described a particular mindset which hindered proactive healthcare-seeking behaviours:

Most from remote areas, they have these non-scientific beliefs. They prioritize it [beliefs] more [than scientific/medical assessments], and then they also have this mentality, “If I’m going to die, so be it”. That’s really part of their culture. (CP015)

In rural communities, faith healers were considered preferable over scientific treatments, and a reliance was placed on traditional therapies (e.g. massage), alongside family support, rather than obtaining healthcare services.

##### Limited stroke knowledge and awareness

Finally, stroke survivors and their families were perceived to lack knowledge and awareness of stroke, healthcare services, financial support, and the importance of rehabilitation. Some stroke survivors were considered unaware that they had even experienced a stroke, while others were suggested to lack any understanding of the importance of rehabilitation. This oblivion meant rehabilitation could be heavily delayed:

I think a big factor is the knowledge or basic knowledge of the patients that after a stroke you still need therapy. Most of the time our patients are just surprised that they have had a stroke for two years and that would be their first time accessing our service. (CP002)

Care providers described how some basic health education was available to stroke survivors (e.g. diet, exercise medication), through mediums such as home visits, public campaigns, and community events. Yet, access could require resources (e.g. transportation), and compared to other needs (e.g. harvest), education was considered low priority and not deserving of such resources.

A widespread issue of misinformation and mistrust in healthcare services was considered problematic. Misleading information on social media (e.g. Facebook, TikTok) was suggested to distort general health literacy: “*things they hear from neighbours and technology is a huge factor. Misinformation is a bit scary because there are others who say, 'eat fat to lose weight … ”'Even we were surprised”* (CP002). Services could also be portrayed as profit-seeking, which was suggested to discourage health-seeking behaviours: *‘” could still hear people trying to discourage those who would go saying, 'they just want to make money off you'”’(CP015).*

#### Theme 3: Desires, needs and opportunities for improvement

This theme illustrates the desires, needs and recommendations of care providers to improve stroke survivors’ experiences of and access to rehabilitation at differing contextual levels.

##### Government and stroke policy action

In recognition of wider, systemic resource constraints, care providers suggested that a national prioritisation of stroke would be necessary to increase funding for stroke services (e.g. infrastructure, technology), as well as financial support for stroke survivors and their families (e.g. PhilHealth for stroke). Care providers felt funding for stroke services would be beneficial for LGUs and barangays who could subsequently ring-fence money for rehabilitation at a local level. In addition, it was suggested that funding should also address inequalities in access to facilities and cost of care. Ultimately, it was felt large national-level efforts were needed to improve stroke services and reduce inequalities:

I think it would require a strong government approach. It’s not just a medical problem, it’s a social problem. As long as the patient is poor, they will struggle to access care. There’s no legislation or any form of support for stroke patients. Here in the Philippines, we are very legislation oriented. If there’s no law for it, it won’t get done. (CP012)

A national prioritisation of stroke was also considered integral to the development and implementation of Philippine-specific stroke rehabilitation protocols and guidelines.

##### Public health messaging

A multi-faceted stroke messaging and education approach, targeting the public, including stroke survivors and their families, was considered necessary to promote stroke literacy at an individual level. Community education events, national public campaigns and active misinformation correction were advocated. Radio was offered as a solution in rural areas and digital and technological solutions including online resources and telehealth were also proposed. Yet digital solutions were only considered helpful in urban areas with technology accessibility and provision:

There’s a trend of patients self-managing, like when you ask, “Why are you taking this?” and they respond, “I saw it on Facebook.” If we could establish a reliable internet self-management program, that might help. And maybe a web-based program created by professionals, like the DOH or an international group. (CP012)

##### Upskilling the community care provider workforce

Care providers described a need for improved rehabilitation, especially at the community level, and preferably with new or improved physical infrastructure (e.g. rehabilitation centres). Local provision and trained care providers were deemed necessary to minimise the impact of financial and geographical stroke care barriers. Therefore, BHWs with their community presence and roles were seen to be important trainees. To enhance the knowledge and confidence of care providers, stroke rehabilitation training was considered necessary. Recommendations for training content included education and the practising of skills, covering topics including what a stroke is, physical therapy, learning exercises and supporting transfers (e.g. from bed to chair), and general health education (e.g. nutrition, medication):

I’d like to learn a more detailed explanation of stroke. Then, what’s the best strategy to quickly assist a stroke patient? Maybe there are techniques we don’t know about. Or, regarding their lifestyle, maybe there are some secrets about medication or daily exercises. For example, if they can’t speak, what’s the best exercise for that? If they can’t move their hands, what should we give them? If they can’t stand, what’s the best technique to help them? (CP010)

Some care providers highlighted interpersonal skills they felt facilitated their interactions with stroke survivors. These skills included patience, sensitivity and empathy and it was felt that these skills should be integrated into training to benefit stroke survivors’ care. Recommendations for training format was group and face-to-face, to both learn physical exercises and watch demonstrations, and foster group discussion and idea exchanges. However, given the large workload of BHWs, the feasibility of training was recognized as a challenge.

##### Providing community support

Furthermore, care providers expressed a need for rehabilitation information to upskill and educate stroke survivors and their families at the community-level. With a desire to empower stroke survivors, some advocated for stroke support groups to give people space to share experiences and support one another. Care providers shared innovations they had seen from stroke survivors including home-made commodes, pulleys and walking aids/supports (e.g. ropes and bamboo) and described the value in programmes, such as community stroke support groups, which provide a platform for sharing such creativity and lived experiences with others:

In my opinion, it’s good to have a platform. Something like, when it comes to therapy … for them to have a voice. They can say what they want … and they can share what they are going through and the success they had after overcoming the stroke. That would be beautiful. (CP005)

## Discussion

### Overview of findings

This paper illustrates findings from one of the first studies to explore the experiences of care providers who work to support stroke survivors and families across different urban and rural settings in the Philippines. Participants described inequitable care, with few established stroke care pathways and an absence of accessible stroke rehabilitation. Barriers to the provision and access of stroke services were characterised across multiple, interconnected levels with national factors impacting community issues (e.g. workforce capacity) and family-level considerations (e.g. stroke awareness). Geographical and financial restrictions were also influential. This study adds to our understanding of the stroke care across the Philippines and provides recommendations to improve stroke services for people in this low-middle-income country both within and beyond the TULAY project which is focused on community stroke care.

### Inequalities in provision and challenges for stroke rehabilitation delivery

As previously recognised [12,16,27,28], inequalities in stroke care infrastructure, pathways and provision remain prevalent across the care provider interviews. Urban areas provided the most comprehensive stroke services, sometimes including rehabilitation [13], whereas most rural settings possessed an absence of public rehabilitation. However, even in urban settings, not all Philippine public hospitals provided stroke services, especially if specialising in alternative medical areas, which replicates previous findings [12,16,27,29]. Care providers’ limited stroke rehabilitation knowledge and skills were further recognised as a challenge. Although some BHWs had some understanding of stroke [30], our findings indicate that this does not always include rehabilitation. This has been recognised by local Philippine government officials who acknowledged the limited number of specialist neurology and rehabilitation staff [16]. Although the government and Stroke Society of the Philippines have developed and implemented initiatives (e.g. prevention and awareness campaigns, professional education), these focus on diagnosis and acute treatment [12,13]. This aligns with care providers’ perceptions that rehabilitation and support for life after stroke is not considered a national priority.

Mirroring previous findings, care providers reported that geographical, environmental and economic barriers furthered hinder stroke survivors’ access to care [13,17,28]. These barriers (e.g. lack of transportation, cost of services, extreme weather systems, and unstable terrain) reflect those also identified by stroke survivors [16]. Stroke survivors and carers reported the financial burden of stroke to be severe, especially when unable to self-fund aftercare and return to work. The introduction of Universal Health Care (UHC) in the Philippines aimed to address these financial inequalities, but to date, its impact has been impaired by a lack of accessible, local healthcare infrastructure [31].

### Implications for a pathway to improve stroke rehabilitation in the Philippines

Improved national prioritisation of stroke, public awareness, workforce infrastructure and community-based rehabilitation were recommended by care providers, aligning with suggestions from local Filipino government officials [17], stroke survivors and their families [16], and a previous review of research in the Philippines [27]. Recommendations and implications for the TULAY project can be divided into multi-level approaches from national policy (top-down) to community level (bottom-up) approaches.

#### Starting from the top: Recommendations for national action

Approaches that align government units and agencies, including the Department of Health, RHUs and LGUs and local leaders (e.g. mayors) have been advocated [17]. Despite the introduction of The Philippine Academy of Rehabilitation Medicine (PARM) guidelines [32], findings suggest there is minimal implementation of stroke rehabilitation guidelines. This may be caused, in part, by the devolved nature of the healthcare system, meaning policies can be ignored or dismissed at a local level [17], or due to lack of care provider awareness. Echoing local government officials, care providers appealed for a strong top-down government approach to mandate the use of policies, and prioritise funding for stroke care, to foster improvements in stroke service [12,17]. This includes making necessary changes to PhilHealth coverage to include comprehensive stroke rehabilitation, and the provision of financial subsidiaries to alleviate the burden of healthcare which would subsequently need to be communicated with stroke survivors and their families [12,27]. Economic recommendations reflect views of stroke survivors and carers who voiced a need for greater access to free medications post-stroke, and accessible public services (e.g. facilities, equipment) [16]. Greater healthcare accessibility could enable stroke survivors to have enough resources for both food and healthcare, rather than one or the other [16], enhancing their quality of life.

Funding allocated to stroke rehabilitation at the local government level could facilitate the education of care providers and the establishment of new rehabilitation facilities. This study highlights the limited capacity of qualified staff to deliver stroke care and rehabilitation in the Philippines, with some rural areas lacking any stroke-aware care providers [13,17,33]. Yet, with dedicated resources for physical therapy and skills training, and incentives for staff in rural areas, such inequalities could start to be addressed.

However, the devolved governance of the Philippine healthcare system and disconnect between national policies and the capacities of local governments [13,17,34] makes a top-down, national government approach challenging. Therefore, recommendations from care providers which can be actioned at community levels present helpful alternative solutions for the TULAY project to action.

#### The needs of the people: Community based rehabilitation as a solution?

Community-based rehabilitation (CBR) has received national recognition for its importance to people living with disabilities in the Philippines [35]. This recognition has prompted the initiation of Community-Based Inclusive Development programmes which aim to bring health services closer to people [36]. CBR has also been found to be of interest to local Filipino government officials, should it be able to improve quality of life, access to care and happiness their communities [17]. Given the localised nature of CBR, it offers a promising avenue to overcome financial and geographical accessibility challenges, and service inequalities, shared by care providers and identified in the wider TULAY project [16]. Proposed components of CBR have included the use of mobile stroke units, stroke rehabilitation training and education for BHWs and care providers across RHUs and LGUs, and educational activities for stroke survivors and their families [12,13,17]. Suggested topics arising from TULAY have included the benefits of rehabilitation [16], together with medical, physiological, psychological, social, financial, and occupational support [17], and culturally grounded coping strategies [16]. However, like stroke survivors, care providers in this study lacked awareness of any stroke-specific community programmes or support groups [16,17]. Telerehabilitation as a form of CBR has shown promise [12,17,37], but as present and wider project findings suggest, this may not be feasible in all rural areas or for those from more deprived socio-economic areas who cannot access electronic devices [16]. Outside of nominal research projects, the operationalisation and implementation of CBR at local levels remains an unexplored endeavour [17].

Individual-level factors may play a role in how individuals engage with health initiatives. The wider TULAY project found that stroke survivors can be passive, with a learned helplessness and a reliance on care providers to ‘fix’ their health conditions [16]. Stroke survivors can also lack health literacy, unaware of the consequences of stroke and the importance of rehabilitation [16], impacting health-seeking behaviours. The Filipino culture of recovery and faith may also inhibit access. ‘Pagtitiis’, described as enduring the difficulty and stress without constant effort in confronting it, reflects individuals’ determination to persevere and can prevent help-seeking action [16,38,39]. Similarly, traditional therapies, including ‘hilot’, massage, herbal remedies, and religious practices comprise culturally dominant coping strategies, which are perceived positively by stroke survivors [16,40,41]. Such culturally embedded concepts, limited health knowledge and helplessness may therefore influence engagement with CBR, even if it is accessible.

Instead, to ensure support meets the needs of stroke survivors, local government officials and care providers in this study have recognised the importance of developing community initiatives together with community groups [17]. These include support groups and self-management programmes to empower and enable stroke survivors to share experiences and learn from others. Such bottom-up strategies resemble participatory approaches to innovation. As one of the world’s fastest growing social media powerhouses [42], the Philippines faces widespread healthcare misinformation, which can generate mistrust of healthcare services. However, through community-led initiatives, giving stroke survivors space to share their needs, priorities and tips with one another, such barriers may be minimised. These participatory approaches harness the ethos of ‘Bayanihan’, a Filipino communal unity and mutuality, which provides a strong foundation to develop locally appropriate, community-driven solutions [16]. Building upon the findings of this paper and the collective evidence, the TULAY project now seeks to co-design culturally relevant and desired stroke rehabilitation support packages for local communities across the Philippines.

### Strengths and limitations

This study recruited care providers from multiple regions and with differing backgrounds (e.g. role), to maximise the diversity and breadth of experiences captured across the Philippines. All interviews were conducted, transcribed and coded by the TULAY Philippine team who are fluent in English and different Filipino dialects (e.g. Tagalog). This enabled contextual nuances to be translated with as much credibility as possible and allowed for participants to use their preferred dialect, maximising their ability to express their views during interviews. This manuscript was co-produced with the TULAY Philippine team to ensure findings are authentic to the interviews and context, thereby providing a trustworthy representation of care providers’ experiences.

However, care providers in this study were self-selecting. Given that some care providers may lack stroke awareness, it may be that only those knowledgeable of stroke chose to participate. Similarly, it may be that those recruited had an agenda and motivation for changes to stroke rehabilitation. The interviews took place following a large-scale national survey of care providers’ thoughts and experiences of stroke care, so it may be that the survey brought stroke care and rehabilitation to the forefront of participants’ attention.

Consistent with small q thematic analysis [23], data collection was anticipated to continue until data saturation. Most topic areas (e.g. barriers to services, types of services, inequalities) did reach saturation. However, when exploring recommendations, it became clear that communities had their own locally derived ideas based on their own geographical location, resources and needs. Due to project budget and time constraints, data collection could not continue until full saturation. However, findings have informed the next phase of the TULAY project, to co-design community-based solutions and self-management support together with and for stroke survivors and household carers.

## Conclusion

Inequalities in stroke services, and specifically stroke rehabilitation, exist across the Philippines. Multiple challenges at national, community, and individual levels are present which relate to stroke policy, the devolved healthcare system, the geographical landscape of the archipelago, financial resources, and cultural norms. Our findings contribute to the growing understanding of stroke service provision and access to care in the Philippines but also shed light on the possible opportunities to overcome barriers and existing inequalities. While national-level changes are needed to mandate policies and improve infrastructure, community-based programmes offer intermediary solutions. Future research should continue to explore opportunities for, and implement, community-level initiatives, to build capacity and knowledge in BHW’s, and meet the needs of stroke survivors in each unique environment, with the aim to enable them to live better following stroke.

## Supplementary Material

Supplementary Files.docx

COREQ checklist.pdf

## Data Availability

The data that support the findings of this study are available from the corresponding author upon reasonable request.
