# Supplementary material for: ‘Wala kaming alam sa stroke care’ (we don’t know anything about stroke care): experiences of care providers delivering stroke rehabilitation care in the Philippines
Source: Glob Health Action. 2026 Jun 5;19(1):2682047. doi: 10.1080/16549716.2026.2682047 (PMC13244504; doi:10.1080/16549716.2026.2682047)
Supplement: Supplementary Files.docx [file ZGHA_A_2682047_SM8817.docx]

**Supplementary File 1: Interview Guide**

Interview topic guide: Care providers (English version) V1.2 - 01/05/2024

Interviewer introduction:

- Introduce self and TULAY Project
- Briefly explain the purpose of the interview and what topics will be covered
- Check how much time the participant has available
- Check they have read and understood the study information – do they have any questions?
- State the interview is voluntary and they can withdraw at any time or request removal of data before it is analysed
- Highlight data protection – data (contact details, interview recording) will be stored securely; personal details will not be shared outside the research team; any names/local places/hospitals you mention will be removed from interview transcripts and reports
- Check consent form has been completed – if not, do this now
- Re-confirm consent for video- or audio-recording the interview (log interview start and end time) and start recording

***Note for interviewers:*** Focus on the questions (middle column) and use the prompts as necessary. Give the participant time to think of their own answer before giving examples.

| **Topic** | **Questions** | **Prompts** |
| --- | --- | --- |
| **EXPERIENCES** | | |
| About you and your role | Could you tell me about your work role and experience of stroke care or rehabilitation? | Title of role  What setting do you work in (inpatient/outpatient/community)?  Region  Urban vs. rural?  What does your work involve?  How many people with stroke do you typically see?  Do you work alone or as part of a team?  How long have you worked with people with stroke? |
| Overview of stroke rehabilitation and care | Can you give a brief overview of stroke rehabilitation and care services in your areas?  How typical is this structure compared to other areas? | What services are available?  Who provides these?  In what settings (e.g. inpatient, outpatient, community)?  Have you worked in another stroke centre before? Describe and compare. |
| **BARRIERS AND ENABLERS** | | |
| Barriers and enablers to access and receipt of rehabilitation (including regional variation and inequalities) | In your experience, what are the major barriers or challenges faced by stroke survivors in accessing or receiving rehabilitation and care?  Do you feel that there are any variations or inequalities in how people access and receive care and support after their stroke?  ***If yes:*** Why? What factors contribute to these variations or inequalities?  How might these challenges or inequalities be overcome? | E.g. lack of knowledge or awareness; lack of facilities (e.g. rehab centres); lack of local support groups; lack of health or social care professionals; lack of family support; lack of PhilHealth coverage/financial capacity  Who is most likely to access and receive rehabilitation?  Geographic differences; urban vs. rural areas; richer vs. poorer areas.  Marginalised populations.  Age; gender; socioeconomic status; education; health status.  Availability of healthcare insurance; policy; transport; availability of health and social care professionals.  E.g. raise awareness; advocacy of primary and secondary prevention; transport; strengthen LGU collaborations with primary health workers; technologies and telerehab; wider PhilHealth availability; online support; self-management programmes. |
| Knowledge, skills and confidence in supporting self-management | Have you received any training in supporting people with stroke? ***If yes:*** Describe the training  What guidelines or protocols for stroke rehabilitation and care do you currently use (if any)? Is there any information that you feel is missing? (knowledge)  What skills do you think are important in supporting people to participate in life after stroke? (skills)  How confident do you feel in supporting people with stroke? What (if anything) would help you feel more confident? (confidence) | Yes/no; type of training; where; when; content; duration.  E.g. national or local guidelines; policies; other sources of information  E.g. encouraging independence; motivating; listening; coaching.  E.g. improved guidance; training; interdisciplinary working; collaboration with stakeholders; use of rehabilitation technologies. |
| Other barriers and enablers to supporting stroke rehabilitation and self-management | Are there any other factors that make it more difficult for you to support people with stroke?  Is there anything we have not discussed that has helped you to support people with stroke? | E.g. financial; time; resources, location – accessibility; patient compliance; motivations |
| **RECOMMENDATIONS** | | |
| General improvements to services supporting life after stroke | Based on your experience, what improvements can be made to the stroke rehabilitation and care systems in the Philippines (in the short or long term)?  Do you know of any examples of innovation or best practice for providing stroke rehabilitation and care within the Philippines?  ***If yes:***   - Why do you think this works well? - Could it be applied to other areas of the Philippines (or other low-middle income countries)? - Why/why not? | E.g. access to healthcare facilities; more patient support groups; training of health workers/village workers; standardised guidance; public awareness; improved funding; inter-, multi- and trans-disciplinary working/exchange of best practices; use of telerehab and rehabilitation technologies; expanded PhilHealth coverage for outpatient and community-based patients and services.  E.g. collaborative programmes; community support groups; online support; telerehab.  E.g. involvement of families/communities/charities/  stakeholders; varied programmes; choice of activities; creative or innovative ideas. |
| Training needs for TULAY programme | We are looking to design some training resources for health and social workers who work with people with stroke and their family members in the Philippines in a community-based setting.  What would you like to see included in these training resources? Are there any particular aspects of supporting people to participate in life after stroke where you would like further training?  What form of training would you prefer? | Content of training  E.g. self-guided learning; taught courses; blended approach; online or in-person; group or one-to-one; peer learning; vocational training |
| Top tips and advice for other care providers | What are your top tips for other care providers who support people to participate in life after stroke? | Providing care within   - Health facilities - Community - Household (working with household carers)   Focus on rehabilitation / participation in life after stroke. |
| Additional comments | Do you have any final thoughts or additional comments? |  |

Interviewer summary:

- Summarise key points discussed during the interview.
- Ask if there is anything they would like to discuss further and state that you are available to discuss the study, especially if there are any issues that arose or discussion points that may have caused distress.
- Check they are still happy for the recorded interview to be used for the purposes stated on the consent form.
- Thank the participant for their time and insights. Give token.
- Ask if they would like to receive a summary of study findings.

**Supplementary File 2: Coding Template**

CP Interviews Codes

a) About CP, the local area and stroke patients

| **Code** |
| --- |
| 1. CP background, role and experience |
| 2. About the area (e.g. region, urban or rural) |
| 3. Lone vs. team working |
| 4. Number of stroke patients and frequency of seeing |
| 5. Perceived causes of stroke in the area |

b) Current stroke services

| **Code** |
| --- |
| Challenges accessing support groups |
| Community support groups, meetings, seminars |
| Diagnosing stroke |
| Home visits and monitoring |
| Hospitals |
| Insurance |
| Making referrals |
| Planning meetings between CPs |
| Provision of (supplies and) medication |
| PS and HC education and training |
| Transport and ambulance services |
| Rehabilitation services provided (including none) |

c) Barriers and enablers to rehabilitation and recovery

| **Code** |
| --- |
| 1. CP factors (knowledge, skills, training and confidence)  Including (lack of) guidelines and protocols; CP knowledge of stroke and rehabilitation; CP training need and availability; CP skills in managing stroke; CP time management; CP attitude and patience; CP communication, rapport and motivation skills; CP confidence; CP support and understanding from family |
| 2. PS and HC factors  Including PS knowledge, awareness and training; HC knowledge, training and supporting PS; media as an enabler or source of misinformation; PS and HC attitude, motivation or willingness; PS and HC finances; PS and HC mistrust of services; PS and HC peer support and encouragement; PS and HC religion and culture; PS and HC transport; PS physical health, mobility and fatigue; HC patience |
| 3. Innovations and strategies by PS, HC and CP  Including importance of being resourceful; anticipating PS needs; home-made commode; home-made walking cane; modified floor for drainage; plastic material on bed (for incontinence); pulleys for bedridden PS; rope and bamboo for physical support |
| 4. Organisational factors (staff and services)  Including availability and retention of stroke rehab staff in barangay (PT OT SP); CP high workload; support from BHWs; support from MHO and doctor to the barrio; continuity of care; availability of healthcare facilities and services; availability of hospital transport; technology and resources |
| 5. Political and financial factors  Including government support (e.g. DoH) including PhilHealth; LGU, RHU and barangay council support (e.g. budget) |
| 6. Geographical factors  Including climate and weather; distance from municipal centre and care services; mountainous terrain |
| 7. Support from the wider community and concerned citizens |

d) Inequities in stroke care and rehabilitation

| **Code** |
| --- |
| 1. Regional differences |
| 2. Urban vs. rural |
| 3. Public vs. private healthcare |
| 4. Perceived reasons for inequities  Including financial; access to transport; differences in climate and weather; family support; PS and HC knowledge and awareness (stroke and services); mistrust of services provided; CP indifference |
| 5. Overcoming inequities |

e) Recommended improvements to stroke services

| **Code** |
| --- |
| Financial and livelihood support for PS and HC |
| Focus on preventive measures |
| Giving people with stroke a voice or platform |
| Improved stroke education and awareness |
| Guidelines and protocols |
| Improved social services |
| Improved stroke and rehabilitation programs (including free rehab); music therapy programs |
| Improved stroke rehabilitation facilities and equipment |
| More medical supplies (inc. medicine for hypertension) |
| More staff specializing in stroke and rehab |
| Stroke support groups |
| Support from NGOs |

f) Top tips and advice for other care providers

| **Code** |
| --- |
| Be polite |
| Have patience |
| Show concern and understanding |
| Be sensitive to culture |
| Don’t give up on patients |
| Time management – give time to stroke patients |
| Educate PS and HC |
| Motivation and encouragement |
| Monitor PS blood pressure and overall health |

g) Training needs and recommendations for TULAY

| **Code** |
| --- |
| 1. Preferred content  Including basic life support and emergency protocol; checking vital signs (e.g. blood pressure); feeding and nutrition; patient handling and personal care; medication management; physical and exercise therapy (inc. mobilization); stroke management; supporting PS to be independent; stroke therapy – general education; how to prevent stroke (e.g. nutrition, lifestyle); information on available services; making use of local resources; mental health and wellbeing for PS; how to care for PS for HC |
| 2. Preferred delivery mode  Including delivered by whom; face-to-face vs. online; individual vs. group |
| 3. Preferred format  Including community seminars or assemblies; digital media (e.g. videos); non-digital (e.g. posters) manual perceived as ‘difficult’; phone call telerehab (offline call); role-playing, group activity |
| 4. Who needs to be trained (e.g. BHWs, HCs) |
